# Supplementary material for: Collagen I promotes cancer cell survival via amino acid import and mTORC1/S6 activation
Source: PLoS Biol. 2026 Sep 18;24(9):e3003555. doi: 10.1371/journal.pbio.3003555 (PMC13614659; doi:10.1371/journal.pbio.3003555)

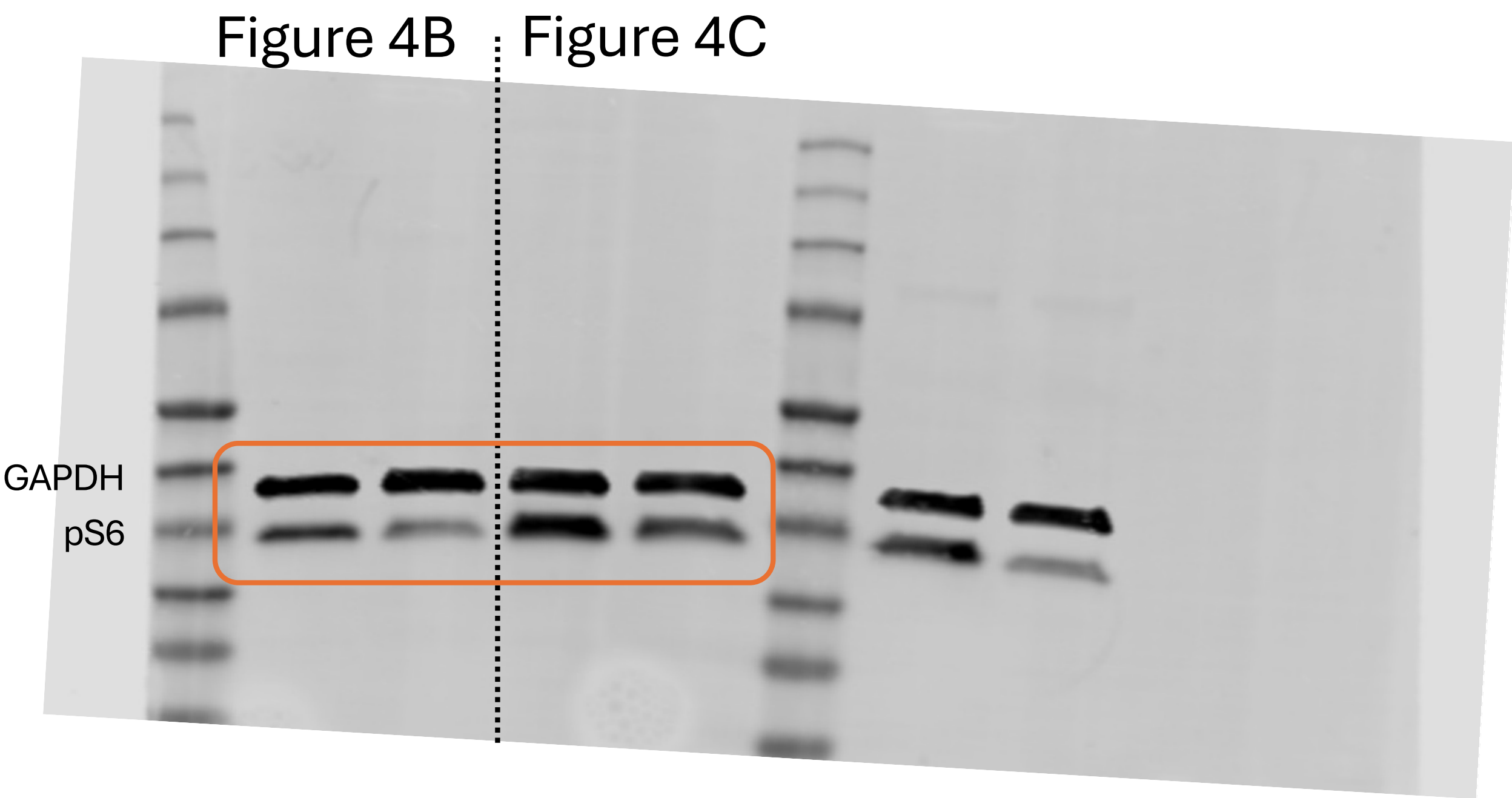
Figure 4B
Figure 4C
GAPDH
pS6

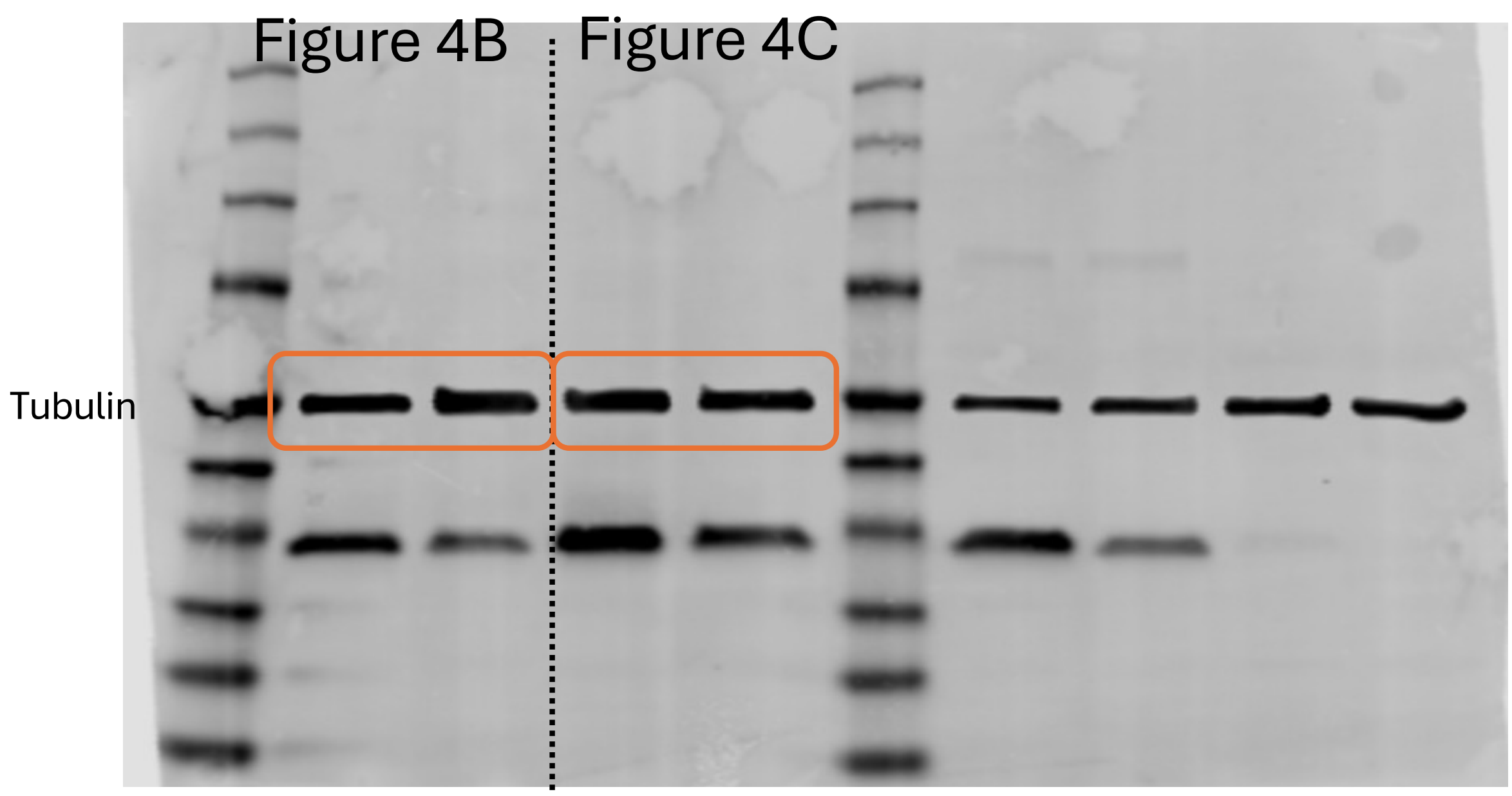
Figure 4B
Figure 4C
Tubulin

Figure 5C

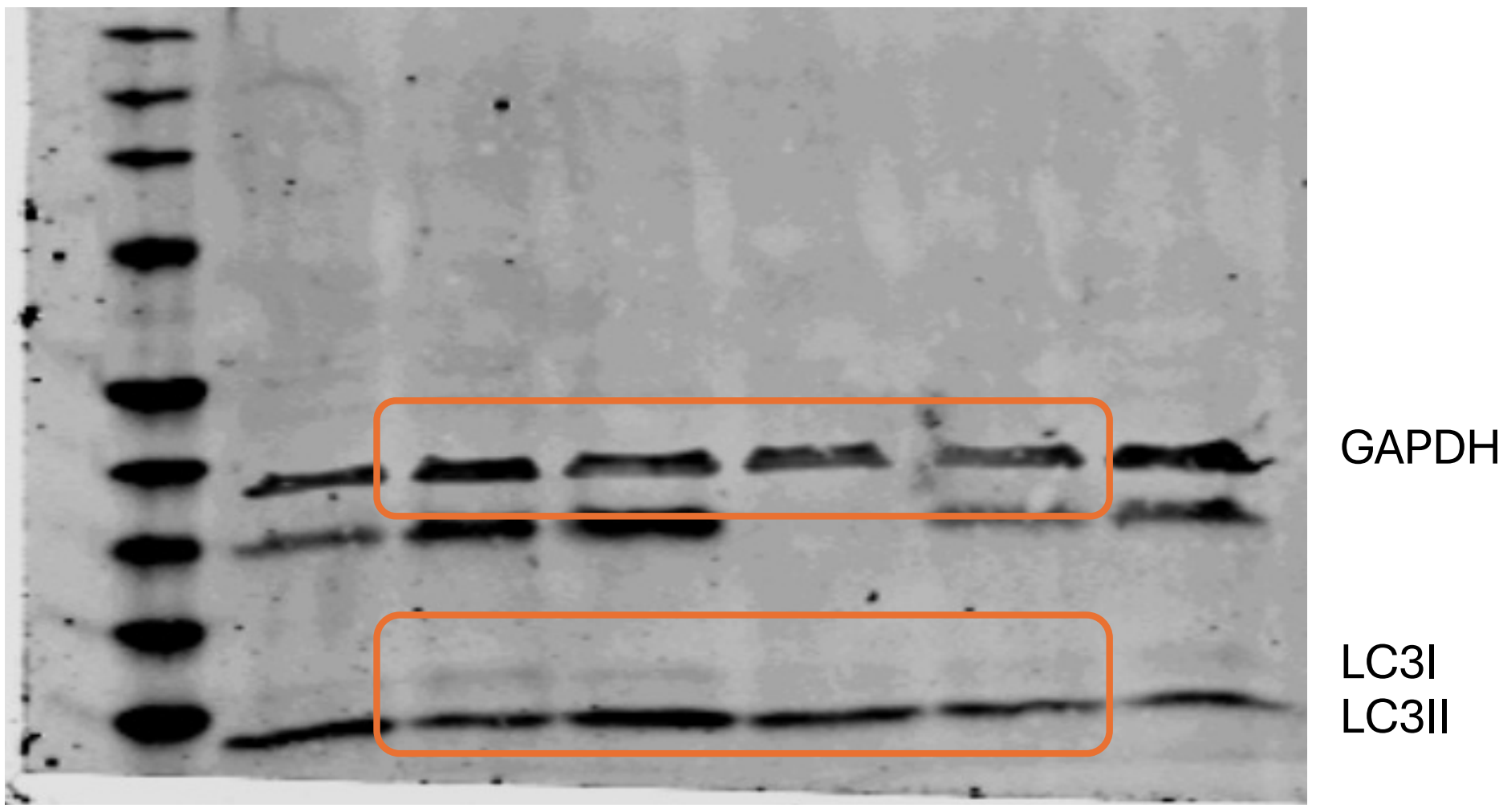

Figure 5D

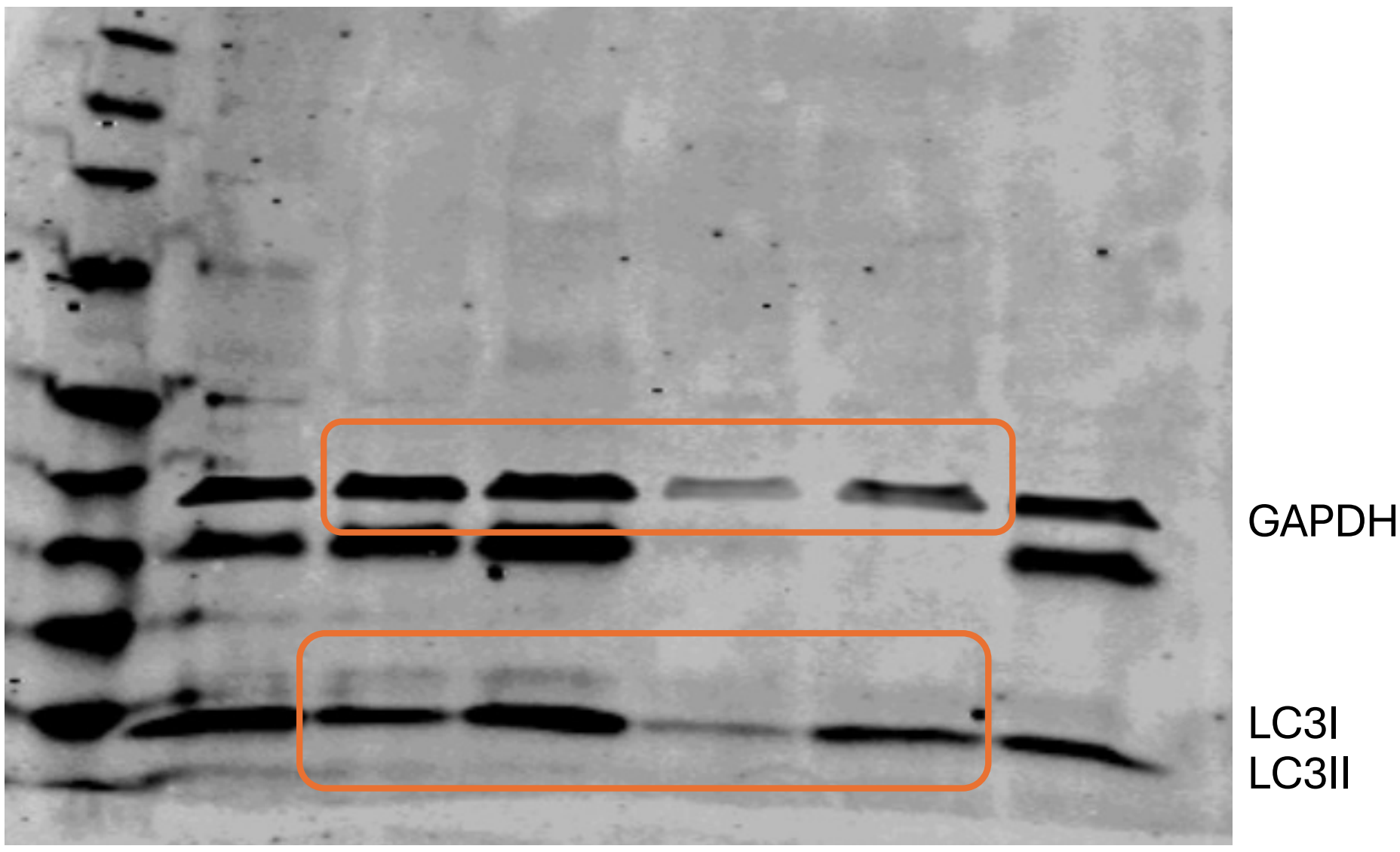

Figure 8A

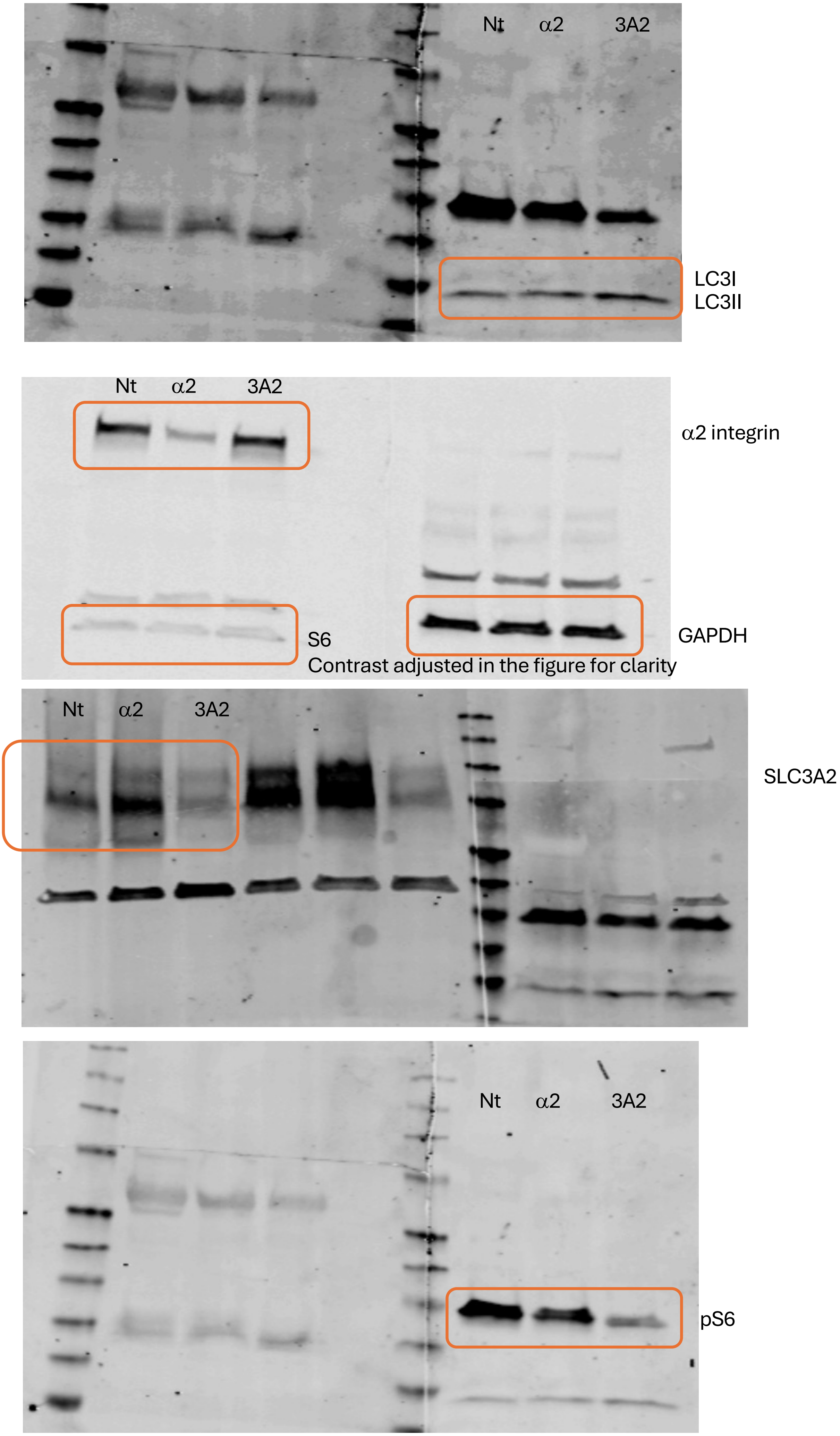
Nt
α2
3A2
LC3I
LC3II
Nt
α2
3A2
α2 integrin
S6
Contrast adjusted in the figure for clarity
GAPDH
Nt
α2
3A2
SLC3A2
Nt
α2
3A2
pS6

Figure 8F Figure 8K

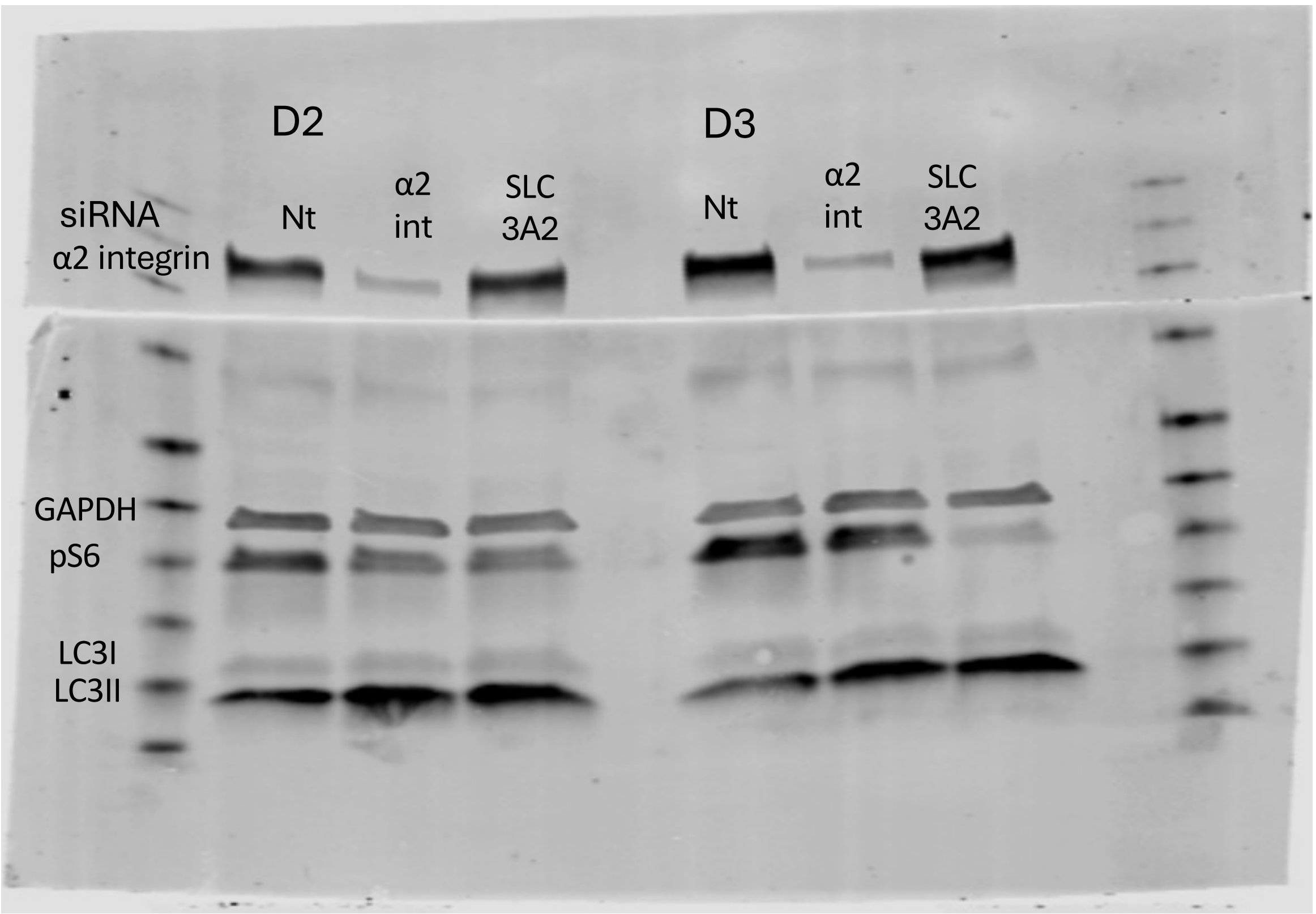

Figure 9E Figure 9J

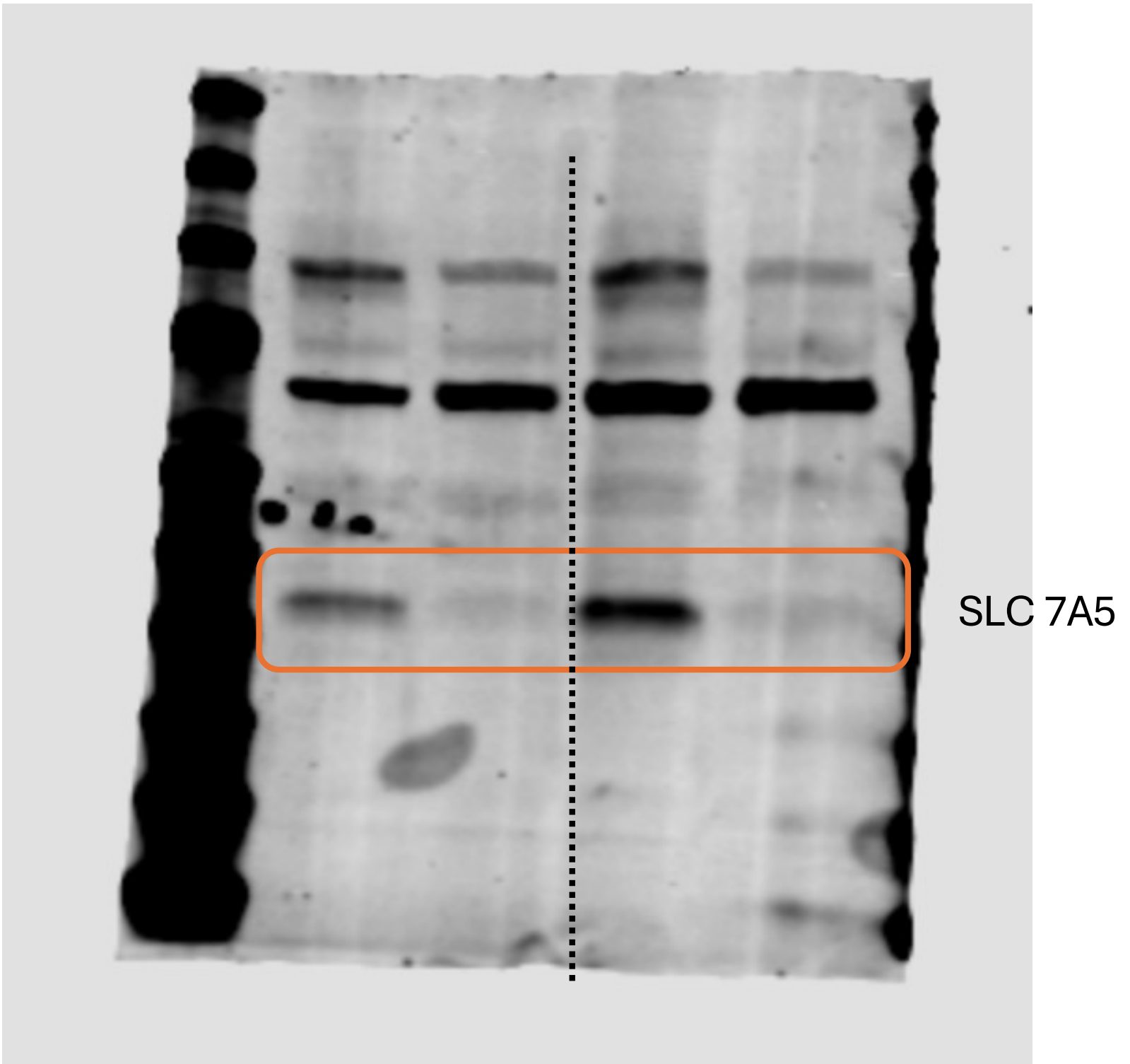

Figure 9E Figure 9J

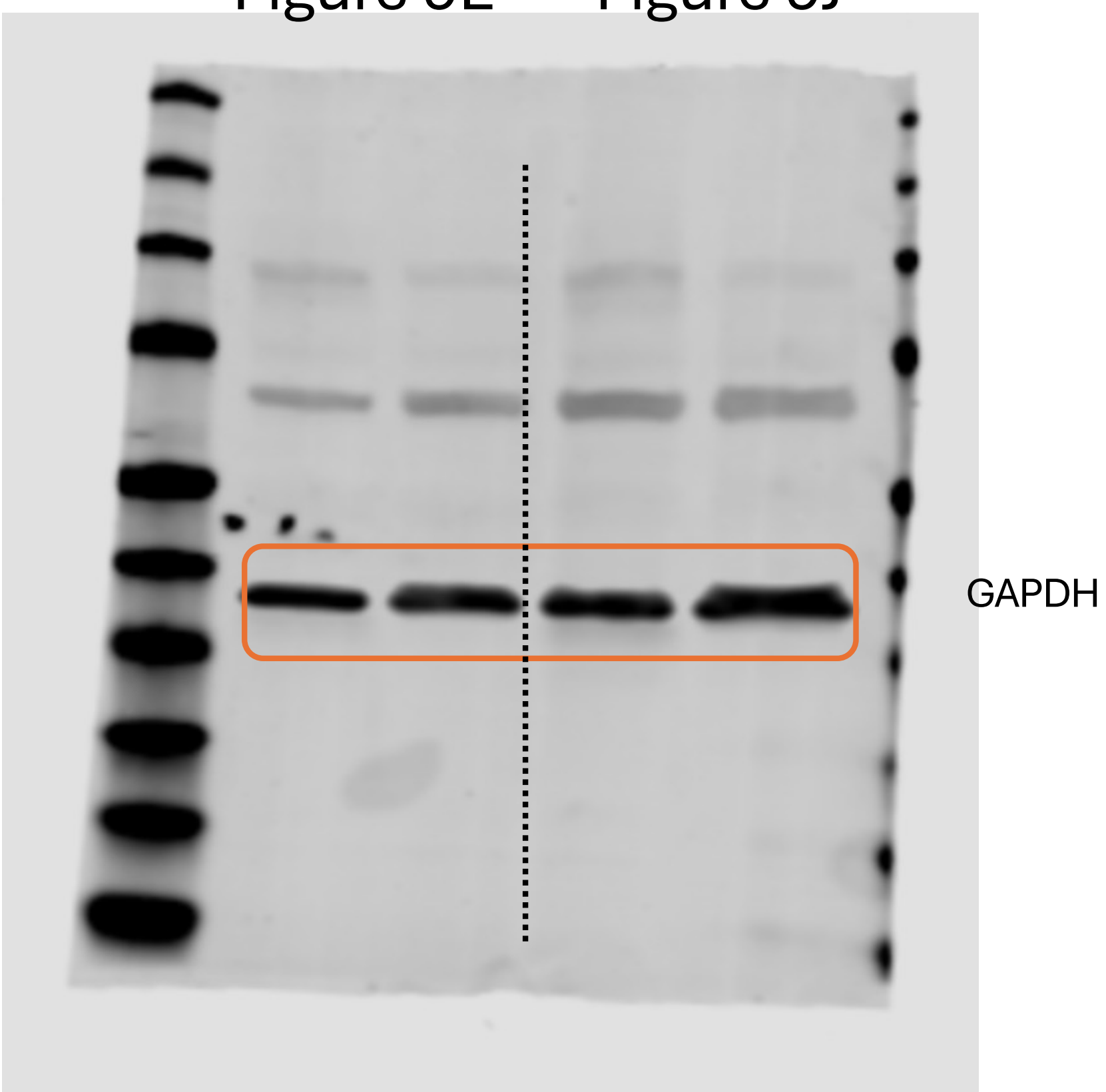

Figure S6C

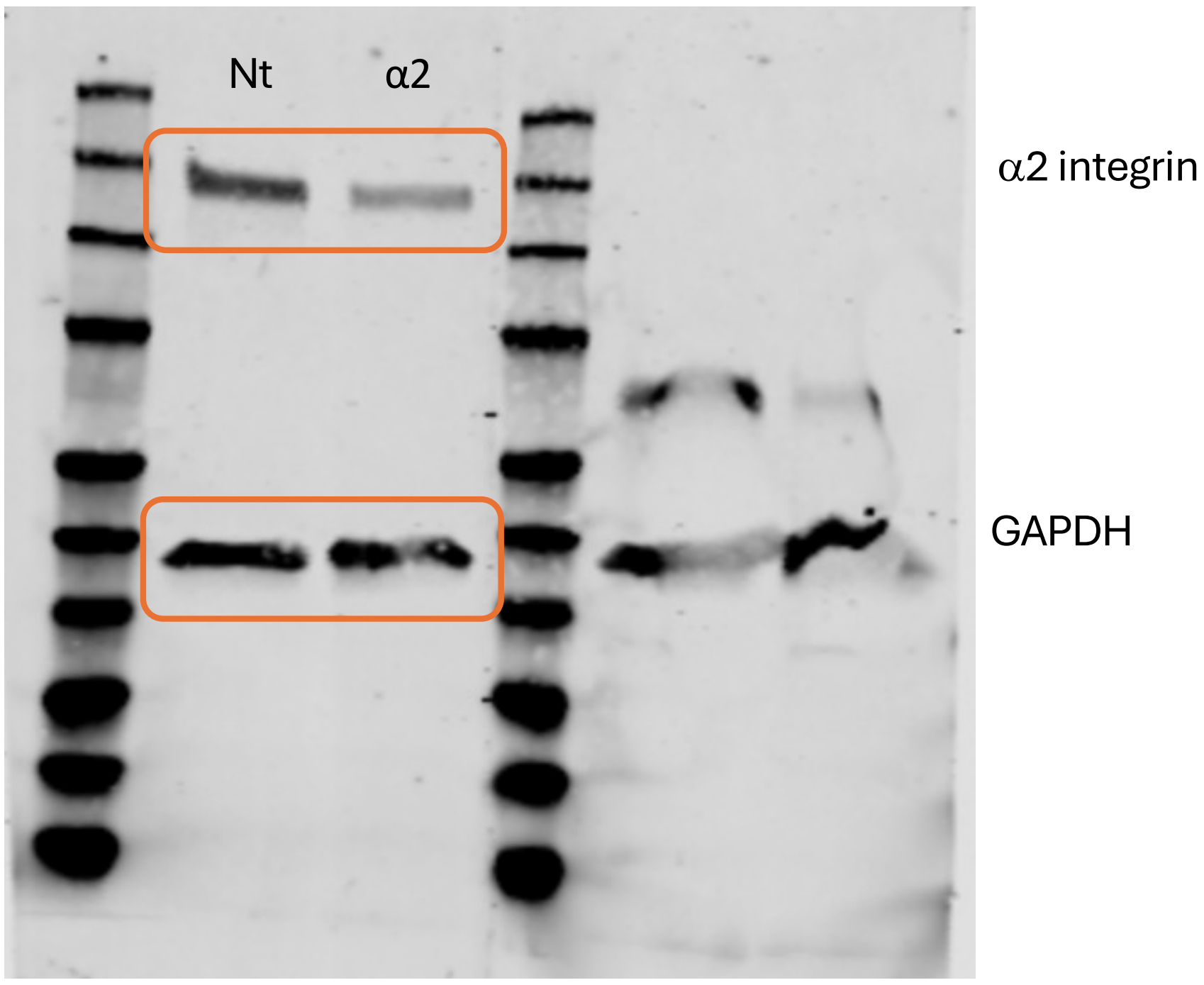

Figure S7A

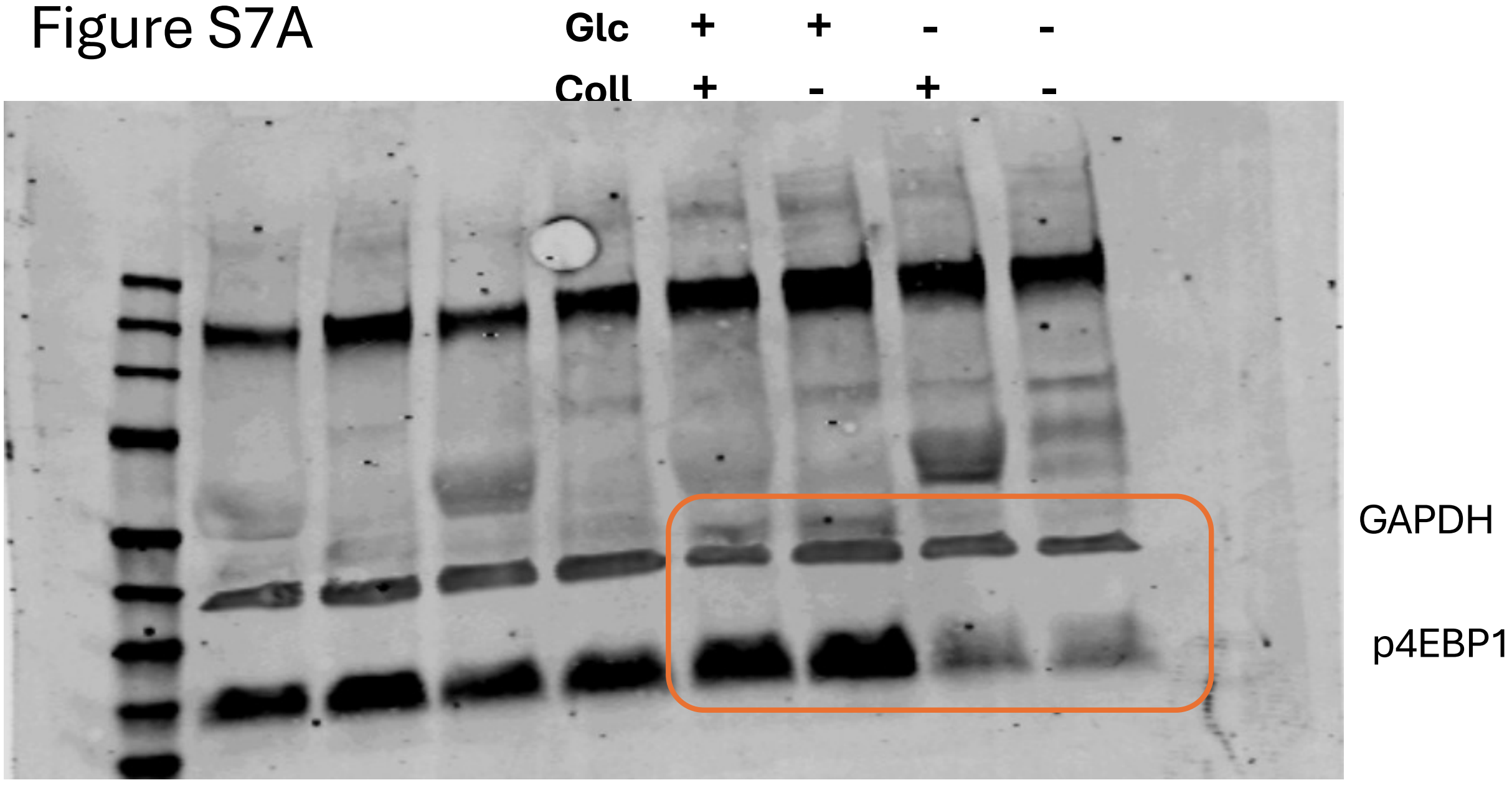

Figure S7B

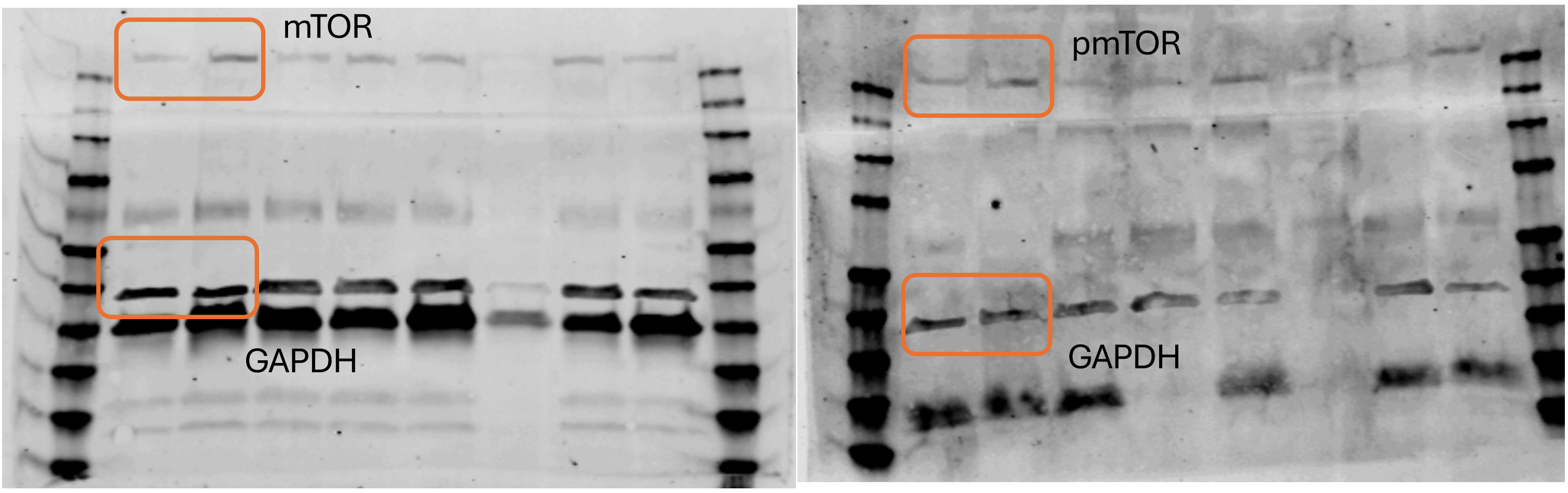

Figure S7C

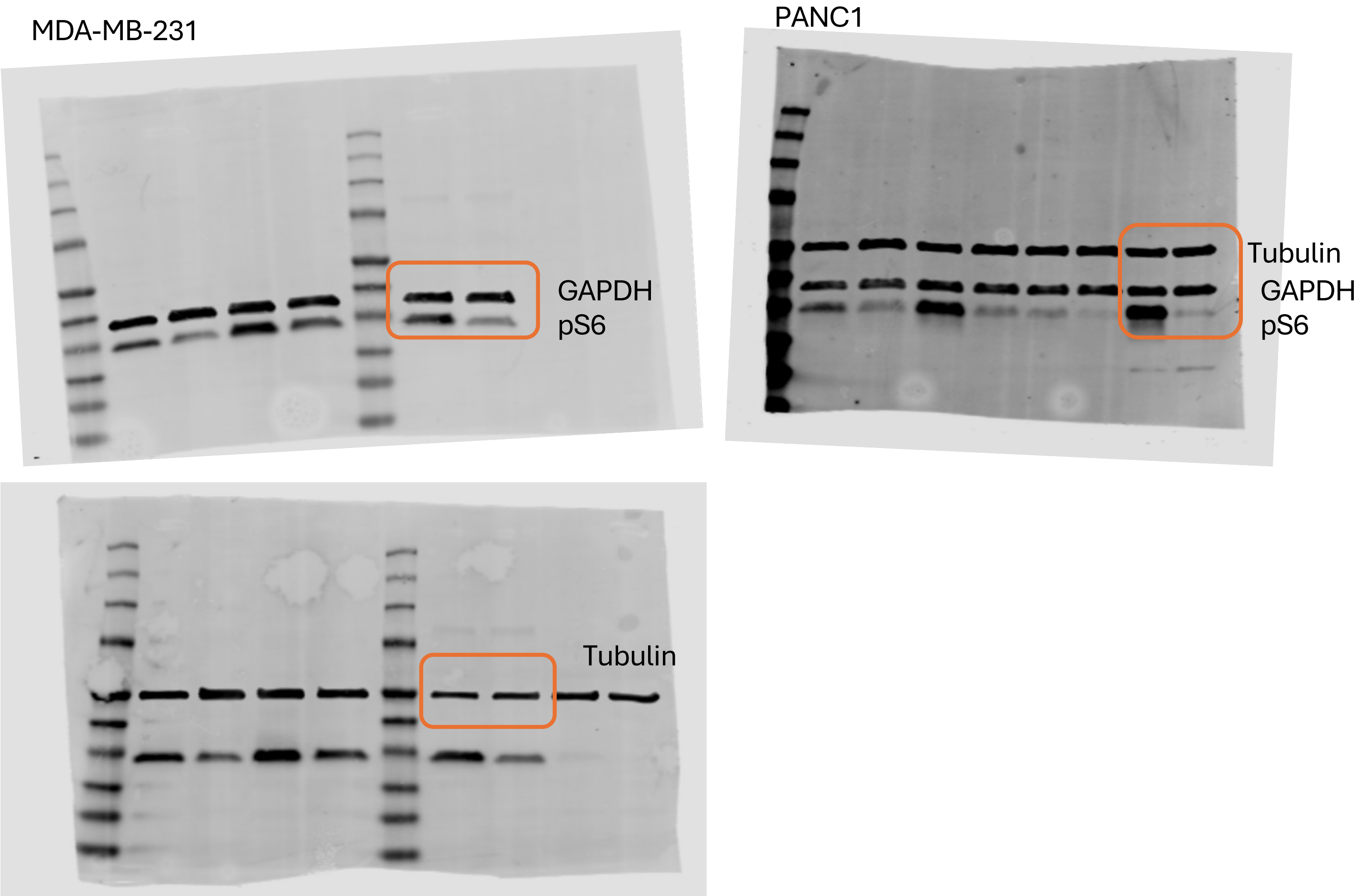

Figure S13

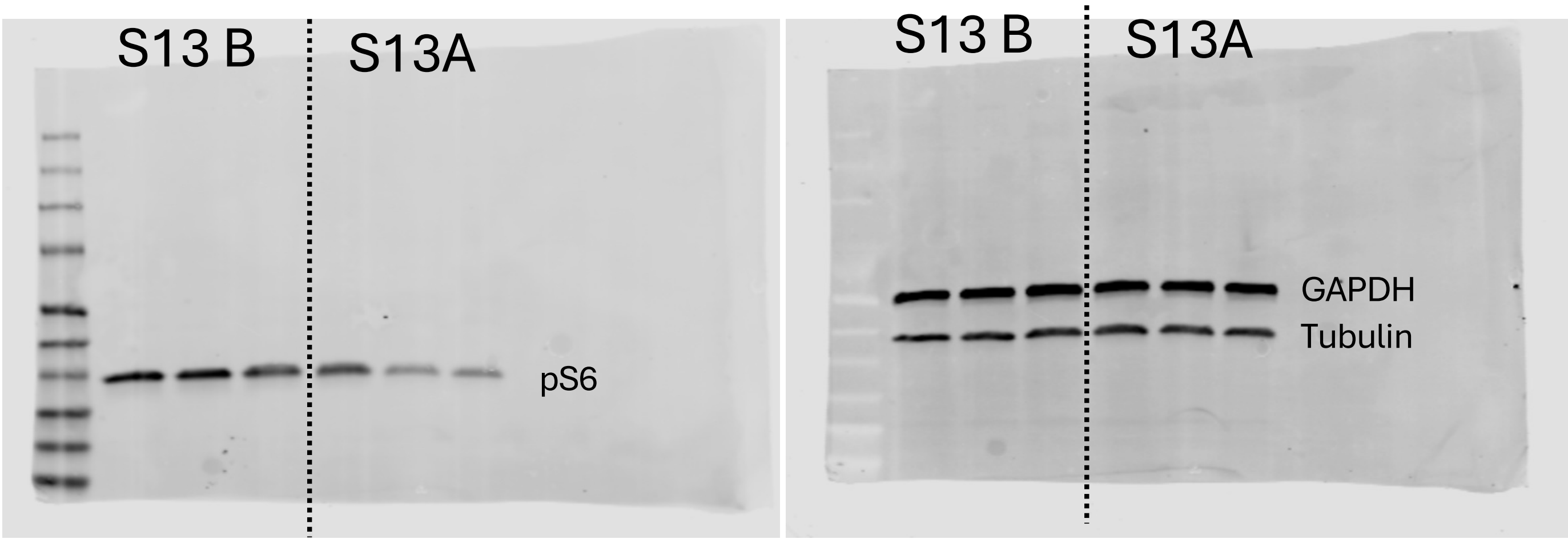

Supplement: Supplementary file 1 [file pbio.3003555.s016.pdf]
