# Supplementary material for: Collagen I promotes cancer cell survival via amino acid import and mTORC1/S6 activation
Source: PLoS Biol. 2026 Sep 18;24(9):e3003555. doi: 10.1371/journal.pbio.3003555 (PMC13614659; doi:10.1371/journal.pbio.3003555)
Supplement: Supplementary file 2 [file pbio.3003555.s041.pdf]

We'd like to thank the reviewers for their constructive comments and feedback, which we believe considerably strengthen our manuscript. We have now addressed all their concerns, as listed below. All the changes in the text are in magenta.

Reviewer 1

The authors' data suggest that the LAT1-4F2hc transporter is critical for the growth of breast and pancreatic cancer cells in vitro under glucose-starved conditions, raising the possibility that this transporter may represent a therapeutic target in vivo. While the presented data support this hypothesis, the manuscript would be significantly strengthened by in vivo validation. Specifically, genetic knockdown or knockout of LAT1-4F2hc, followed by assessment of tumor growth in preclinical models using the same cell lines employed in vitro, would provide more compelling evidence for its role in tumor biology.

We agree with the reviewer that this would be an important point, however we are not in a position to carry out these experiments within the time frame of this revision. However, to support the role of LAT1 in tumorigenesis, we expanded our discussion to include evidence that, in mouse model of colorectal cancer, Slc7a5 deletion was found to prevent tumour growth and metastasis. Consistently with our findings, this was coupled with a reduction in S6 phosphorylation (but not 4EBP1) and an increase in autophagy. Interestingly, Slc7a5 expression was found to be upregulated by Kras mutation.

One limitation of the current experimental design is that tumor cells in vivo experience deprivation of multiple nutrients simultaneously, rather than glucose alone. Although the in vitro glucose-starvation system is valuable for isolating glucose-specific effects, it may not fully capture the complexity of the tumor microenvironment. It would therefore be informative to determine whether the reported alterations are specific to glucose deprivation or whether similar changes in amino acid import and mTOR signaling occur under additional nutrient-limiting conditions, such as reduced serum and/or growth factor availability.

We are now including new data in **S12 Fig**, showing the response of breast and pancreatic cancer cells to serum deprivation. The presence of collagen I supported the growth of MDA-MB-231 (**S12A Fig**), while it reduced the proliferation of PANC1 cells (**S12B Fig**). Mechanistically, collagen I supported the phosphorylation of S6 (**S12C Fig**), downstream of mTORC1, but did not affect essential amino acid intracellular levels (**S12D Fig**). This suggests that the regulation of LAT1-mediated amino acid transport is not a general response to nutrient limitation.

For the BTT-3033 and siRNA experiments, inclusion of data from non-glucose-starved control conditions would be very helpful. Such experiments would clarify whether the observed effects reflect activation of a stress-induced pathway or a constitutively active mechanism independent of glucose deprivation. In particular, while the results suggest that α2β1 integrin modulates cell growth on collagen under glucose-starved conditions, it remains unclear whether similar effects would be observed under nutrient-replete conditions.

New **S4 Fig** shows the cell proliferation experiments conducted in complete media in the presence of BTT-3033 and integrin downregulation, both on plastic and collagen I. Overall, we detected a more profound inhibition of cell growth in PANC1 compared to MDA-MB-231 cells, and a stronger effect in the presence of collagen I compared to plastic. The data indicated that $\alpha 2\beta 1$ integrin controls cell growth regardless of nutrient availability. We then assess the impact of $\alpha 2\beta 1$ inhibition on the phosphorylation of S6 (pS6). While BTT-3033 resulted in about 50% reduction in pS6 under amino acid starvation (**S13A Fig**), it did not affect S6 phosphorylation in

complete media (**S13B Fig**), indicating that α2β1 specifically promoted S6 phosphorylation under glucose starvation.

Several specific points also warrant clarification:
* *Figure 4D: In the bar graph, is the decrease in LC3-II intensity following BTT-3033 and rapamycin treatment statistically significant? This should be clearly indicated*.

BTT-3033 and Rapamycin treatments were performed on collagen I. None of the additional differences are statistically significant. We have now labelled them 'ns' in the figure, which is now new **fig 5C,D**.

** Figure 5: Providing the raw metabolic data values represented by the red dots in panels B and E as a supplemental Excel table would improve transparency. The same applies to Figures S8B and S8D*.

The raw metabolic data will be available on the ORDA database upon publication of the paper.

** Figure 2: Please explicitly state in the main text that the cells were glucose starved in this experiment. Although this is implied by the figure legend title, inclusion in the text would improve clarity for readers*.

Reference to glucose starvation has now been included in the text.

* *Page 5, line 1: The statement that α2β1 is the "main" collagen receptor is not sufficiently supported, given the number of known collagen I receptors. A more precise phrasing, such as "one of the most well-characterized collagen receptors," would be more appropriate*.

This has now been changed.

** Page 20, Methods section - Starvation conditions: The glucose-starvation conditions are described as the use of glucose- and pyruvate-free medium supplemented with 10% dialyzed FBS. This approach suggests that, in addition to glucose deprivation, the removal of pyruvate and other small molecules normally present in serum could also contribute to the observed phenotypes. However, throughout the manuscript, the experimental condition is described primarily as "glucose starvation," which may oversimplify the nature of the nutrient deprivation. The authors are encouraged to clearly define what they mean by glucose starvation early in the main text and to acknowledge that deprivation of additional metabolites may also influence the observed responses in these experiments*.

We have clarified the lack of pyruvate in our starvation conditions. In all experiments, complete media was in the presence of dialysed FBS as well and we did not detect any change in cell growth when comparing complete media with 10%FBS or 10% dialysed FBS.

Reviewer 2

1. Cell growth assays have inconsistent timepoints. Growth curves start at different days depending on the cell line (i.e., MDA MB 231 measured from D2 and PANC1 starting from D0). D2 time points already show significant differences between collagen and plastic, hard to discern whether this is due to inconsistent plating at D0 or a result of increased proliferation. Include D0 measurements. It would also be beneficial to include Complete media conditions here, to see how much collagen rescues proliferation under Glc deprivation.

We apologies for the inconsistency. We have now repeated these experiments, and we are presenting D0 data, consistent time points in both cell lines and the complete media for comparison (**Fig 1A-D**).

2. At present, the data show several correlations, but these are weakened by inconsistencies in experimental design that make the data difficult to interpret. Authors show that Collagen increases pS6 and a2B1 inhibition reduces pS6, they also show that this is not the case for 4E-BP1, but there is no actual demonstration that S6 phosphorylation is occurring downstream of mTORC1. Blocking mTORC1 with rapamycin/Torin1 will help confirm this. I also strongly suggest assessing mTORC1 using western blots.

To strengthen the link between mTORC1 activation and ECM-driven cell proliferation under glucose starvation and provide further mechanistic information, we have measured the impact of mTORC1 inhibition on cell proliferation. New data in **fig 4D** shows that Rapamycin treatment significantly reduced the growth of both breast (MDA-MB-231) and pancreatic (PANC1) cells proliferation on collagen I under glucose starvation. We include new western blot data showing that S6 phosphorylation is increase on collagen I compared to plastic under Glc starvation (**Fig 4B,C**) and this is opposed by Rapamycin treatment (**S7C Fig**), while mTOR phosphorylation is not affected by the presence of collagen I (**S7B Fig**).

3. LAT1 mediated amino acid uptake is not directly demonstrated. They show that there is an increase in amino acid pools on collagen compared to plastic under glucose deprivation. LAT1 subunits show increased surface staining, and knockdown reduces intracellular amino acids. These are measurements of steady-state pools of amino acids, and do not reflect transport rates. While, I am happy to see the authors assessed the contribution of autophagy to these pools, this accumulation could be a result of reduced utilisation due to low proliferation and reduced protein synthesis (likely the case under Glc deprivation). Authors should show increased transport with radiolabelled or stable-isotope labelled AA uptake assays over a dynamic range of time and block LAT1 pharmacologically with a specific inhibitor such as JPH203 for example, or by knockdown. 13. Should prove that D-Phe does block LAT1 transport using an uptake assay.

We have now included new data (**S12Fig**) showing that D-Phe significantly reduced 13C-Tyrosine import.

4. Should co-stain SLC3A2 and SLC7A5 to show that they are co-localised rather than immunostaining individually. Furthermore, it would be good to include complete media conditions here, it seems SLC3A2 is not being trafficked to the membrane efficiently, perhaps due to reduced N-glycosylation as a result of reduced Glc availability.

Unfortunately, both antibodies are generated in rabbit, therefore we are not able to perform co-localisation studies.

5. LAT1 localisation and amino acid levels are assessed independently of a2B1 manipulation, unclear how this is specific to collagen under Glc deprivation. Would be beneficial to assess whether a2B1 inhibition alters LAT1 membrane localisation and to assess whether amino acid uptake is altered.

We are now including metabolomics data showing that treatment with BTT-3033 did not affect intracellular essential amino acid pools in both MDA-MB-231 (**Fig 6G**) and PANC1 cells (**Fig 6H**).

Consistently, BTT-3033 did not affect 13C-Tyrosin import and resulted in a small reduction in LAT1 membrane localisation in MDA-MB-231 cells, but not in PANC1 cells (**S11 Fig**).

6. SLC3A2 knockdown is not a LAT1 specific perturbation. As mentioned by the authors, SLC3A2 is important for different amino acid transporters including xCT (which is very important under glucose deprivation and potentially here considering Gly/Ser metabolism is a top hit in all untargeted metabolomics studies). Should use SLC7A5 knockdown for mechanistic claims. 7. mTORC1 dependence on LAT1 effects is not tested, they show that SLC3A2 knockdown reduces pS6, but do not test whether LAT1 inhibition reduces collagen-induced mTORC1 activation. 14. Does D-phe block mTORC1 activation in presence of Coll + Glc deprivation like a2 inhibition?

We have now confirmed the role of LAT1 in mTORC1 activation by showing that treatment with D-Phenylalanine significantly reduced S6 phosphorylation (**S13 Fig**).

9. Overstatements such as "this implies the presence of collagen I alleviates the metabolic stress induced by Glc deprivation enabling cells to maintain an anabolic state".

We understand the reviewer's point that the sentence (hypothetical in our intentions) could be perceived as an overstatement of our findings, and it has now been removed.

10. There is a lack of complete media controls throughout the entire study. I believe these important to understand just how much collagen is protecting cells from glucose deprivation. Similarly in some places where claims are made about collagen there isn't a plastic control.

We have now included the complete media condition and the plastic control throughout the manuscript.

11. You state that "a2B1 integrin was required for cancer cell growth and invasion under Glc starvation" but you show none of these results under complete media.

New **S4 fig** shows the cell proliferation experiments conducted in complete media in the presence of BTT-3033 and integrin downregulation, both on plastic and collagen I. Overall, we detected a more profound inhibition of cell growth in PANC1 compared to MDA-MB-231 cells, and a stronger effect in the presence of collagen I compared to plastic. The data indicated that $\alpha 2\beta 1$ integrin controls cell growth regardless of nutrient availability. We then assess the impact of $\alpha 2\beta 1$ inhibition on the phosphorylation of S6 (pS6). While BTT-3033 resulted in about 50% reduction in pS6 under amino acid starvation (**S13A Fig**), it did not affect S6 phosphorylation in complete media (**S11B Fig**), indicating that $\alpha 2\beta 1$ specifically promoted S6 phosphorylation under glucose starvation.
We have recently published that $\alpha 2\beta 1$ is required for the migration and invasion of breast, ovarian and pancreatic cancer cells (Martinez et al., 2024), the aim here was to show that this was also the case under glucose starvation. We now highlighted this more clearly in the text.

12. Figure 7 you show that the a2 and 3A2 KD reduce S6 but you should include the plastic conditions to show that there is first an increase in pS6 and then this reduced to the plastic/complete media conditions. 8. Should assess mechanistic claims within short term Glc starvation to avoid the effects of long-term glucose starvation.

New data show the increase in S6 phosphorylation by Western Blotting on collagen I compared to plastic, after 2hr glucose starvation (**Fig 4**). Moreover, pharmacological inhibition of $\alpha 2\beta 1$

integrin and SLC7A5 significantly reduced pS6 levels on collagen I under glucose starvation, but not in complete media (**S13 Fig**).

15. Doesn't seem to be any collagen specific differences in LAT1 requirement, equally required in complete and Glc free media and differ by cell line, but the caption states "SLC3A2 and SLC7A5 were required for collagen I-dependent cell growth in 2D and 3D systems." Should show differences between collagen and plastic.

We rephrased the caption.

Minor comments:
- Figure 8F and 8J are duplicated and I can see that the Nt si appears the same in both experiments, this should be highlighted in the caption for Figure 8.

We apologise for this mistake, we have now replotted the graphs with the correct Nt si, which have not been duplicated.

- Validate rapamycin effectiveness in figure 4C/D. Should have downstream targets of mTORC1 from the same lysates to show that rapamycin reduces pS6.
- Is the Rapamycin column in figure C D supposed to be with collagen? To see whether autophagy is increased to plastic levels? Currently "-".

New **S7 Fig** shows the effect of Rapamycin in inhibiting S6 phosphorylation. Yes, it is on collagen I, the figure has been corrected.

- GAPDH is not a good loading control particularly for metabolic studies as it is influenced by glycolysis and ROS, I recommend replacing these with a different loading control or using total protein staining/ponceau.

All the new western blots have been performed including GAPDH and Tubulin. We did not detect any difference between GAPDH and Tubulin, suggesting that, at least in our system, they are both valid as loading control (**Figure R1**).

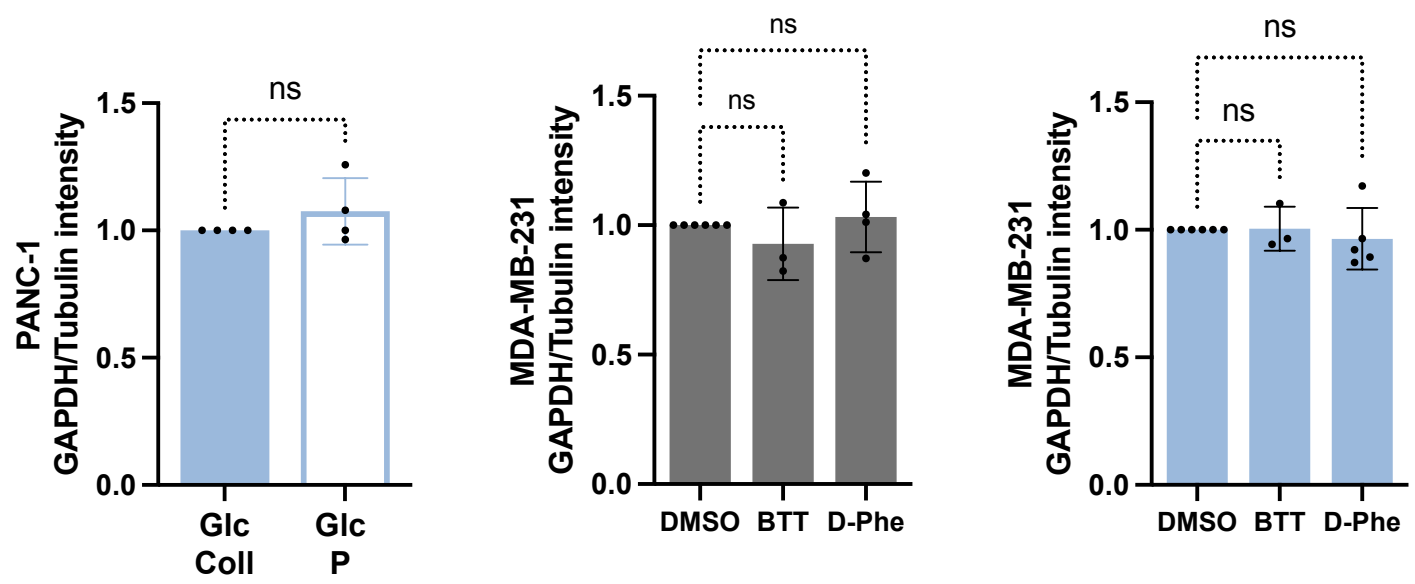

Figure R1. GAPDH/Tubulin ratio is not affected by the experimental conditions used in this study. (A) PANC1 cells were seeded on 2mg/ml collagen I Coll) or plastic (P) in Glc deficient media for 1 day. (B,C) MDA-MB-231 cells were grown on 2 mg/ml collagen I for one day, and starved in Glc-free media (C) or kept in complete media (B) for 2hr, in the presence of 10μM BTT-3033 (BTT), 50mM D-Phenylalanine (D-Phe) or DMSO (Ctrl). Lysates were collected and the levels of phosphorylated Tubulin and GAPDH were measured by Western blotting. Data are presented as mean ± SEM, N≥3 independent experiments.

- It is hard to understand what cell line some figures are referring to at a glance, would be helpful to clearly label figure panels.

All graphs have now been labelled with the name of the cell line on the Y axis.

- Please label heatmap scales from metabolomics studies, unclear what they represent.

This has now been included in the figure legend.

- Comparative conditions should be on the same graph, Glc starvation and Complete media on different graphs with different scales for Figure 8a/b.

We made all graphs with the same axis range.
