# Supplementary material for: Collagen I promotes cancer cell survival via amino acid import and mTORC1/S6 activation
Source: PLoS Biol. 2026 Sep 18;24(9):e3003555. doi: 10.1371/journal.pbio.3003555 (PMC13614659; doi:10.1371/journal.pbio.3003555)
Supplement: Supplementary file 3 [file pbio.3003555.s042.pdf]

## Reviewer 1

*No additional concerns*.

## Reviewer 2

*1. I previously requested a more direct demonstration of LAT1-mediated AA transport because the targeted metabolomics experiments measure "steady-state" intracellular amino acid pools, which can reflect changes in transport, metabolism, protein synthesis, etc. I appreciate that the authors have included a dynamic 13C-Tyr uptake exp using D-Phe. However, the reduction in 13C-Tyr uptake is relatively modest, making it difficult to conclude that LAT1 import is efficiently blocked. Furthermore, the uptake assay does not directly compare AA transport between collagen vs. plastic or complete vs. Glc-starved conditions, which is central to the proposed model. While SLC7A5 KD demonstrates that LAT1 contributes to intracellular AA levels it again is not compared to complete media conditions, is there actually increased import relative to complete conditions in Coll-I seeded cells or is it maintained? Therefore, I do not think the current data fully support that collagen I promotes EAA import under Glc- starvation. Either stronger transport experiments should be provided (i.e., using a more selective LAT1 inhibitor like JPH203, or additional substrate competition rescues with D-Phe) with the necessary control conditions, or conclusions should be moderated throughout the manuscript to state that coll I increased intracellular EAA abundance (if complete media + Coll I metabolite experiments are done). Additionally, can the authors clarify what the targeted metabolomics experiments are normalized to.*

We have now moderated to conclusions throughout the manuscript. Targeted metabolomics data normalisation has now been included in the method section.

*2. Conclusions regarding mTORC1 activation should be revisited. The additional rapamycin experiments strengthen the conclusion that collagen-induced S6 phosphorylation is largely mTORC1-dependent. Therefore, there are some statements throughout the manuscript that refer to "mTORC1 activation" that should be changed to RPS6 phosphorylation specifically since p70S6K1 and 4E-BP1 were not altered in Coll I conditions. Furthermore, the conclusion that "mTORC1 signalling was required for ECM-dependent cell growth under Glc-" is not fully supported by the current experiments. Figure 4D/E only examines Rapa treatment in collagen-grown Glc-starved cells and not complete medium and on plastic, therefore, it doesn't demonstrate that it's required for ECM-dependent growth. Including the corresponding controls would clarify how much of the collagen phenotype is mTORC1-dependent.*

This has now been changed as suggested.

*3. Mechanistic interpretation of SLC3A2 KD should be moderated. The manuscript states that SLC3A2 KD demonstrates that "LAT1-4f2hc transporter mediated coll-I dependent mTORC1 activation and autophagy inhibition". SLC3A2 is not specific to LAT1 so SLC3A2 KD results cannot be attributed solely to LAT1 activity. I recommend moderating these statements relating to Fig 8 unless experiments are repeated with the SLC7A5 KD cells generated in Fig 7H,I.*

We moderated the statement, as requested.

*4. The targeted metabolomics performed after 4 days of Glc starvation should be interpreted cautiously. By this stage, Fig 1 demonstrates substantial cell death in cells grown on plastic, whereas Coll-I grown cells remain largely viable. Consequently, metabolic differences may*

*reflect differences in cell survival rather than metabolic differences. It would also be helpful to include the Day 1 and Day 2 cell death quantification for these cell lines (Fig 1E), as these correspond to the time points analysed in Figures 6G/H.*

We have now mentioned this point in the description of the targeted metabolomics time course. We did not perform cell death quantification at day 1 and day 2.

Minor comments:
*- Why were spheroid experiments performed for different durations (2 days for MDA-MB-231 vs. 6 days for PANC1)? A brief justification would be helpful*.

This is due to the difference in invasiveness between the 2 cell lines. At day 2, there is no invasion in PANC1 cells, while at day 6 MDA-MB-231 spheroids are impossible to image, as there is too much invasion. We have now mentioned this point when describing the results.

*- Please include WB validation for SLC7A5 KD efficiency.*

We now included Western Blot analysis showing the SLC7A5 KD efficiency in both MDA-MB-231 and PANC1 cells (new Fig 9E,J).

*- For the serum starvation experiments (Figure S15), was the ISR activated to a similar extent as during Glc starvation? In addition, how was the 8-day serum starvation timepoint selected?*

We did not access ISR activation under serum starvation. Day 8 was selected as it resulted in a statistically significant increase in MDA-MB-231 cell numbers on collagen I compared to plastic.
