# Supplementary material for: Collagen I promotes cancer cell survival via amino acid import and mTORC1/S6 activation
Source: PLoS Biol. 2026 Sep 18;24(9):e3003555. doi: 10.1371/journal.pbio.3003555 (PMC13614659; doi:10.1371/journal.pbio.3003555)
Supplement: Peer review history

# Editor report: Decision Letter 0

**DOI:** 10.1371/journal.pbio.3003555.r001

**Authors:** Ines Alvarez-Garcia

20 Nov 2025

Dear Elena,

Thank you for submitting your manuscript entitled "Collagen I promotes cancer cell survival via amino acid import and mTORC1 activation" for consideration as a Research Article by PLOS Biology.

Your manuscript has now been evaluated by the PLOS Biology editorial staff as well as by an academic editor with relevant expertise and I am writing to let you know that we would like to send your submission out for external peer review.

However, before we can send your manuscript to reviewers, we need you to complete your submission by providing the metadata that is required for full assessment. To this end, please login to Editorial Manager where you will find the paper in the 'Submissions Needing Revisions' folder on your homepage. Please click 'Revise Submission' from the Action Links and complete all additional questions in the submission questionnaire.

Once your full submission is complete, your paper will undergo a series of checks in preparation for peer review. After your manuscript has passed the checks it will be sent out for review. To provide the metadata for your submission, please Login to Editorial Manager (https://www.editorialmanager.com/pbiology) within two working days, i.e. by Nov 24 2025 11:59PM.

If your manuscript has been previously peer-reviewed at another journal, PLOS Biology is willing to work with those reviews in order to avoid re-starting the process. Submission of the previous reviews is entirely optional and our ability to use them effectively will depend on the willingness of the previous journal to confirm the content of the reports and share the reviewer identities. Please note that we reserve the right to invite additional reviewers if we consider that additional/independent reviewers are needed, although we aim to avoid this as far as possible. In our experience, working with previous reviews does save time.

If you would like us to consider previous reviewer reports, please edit your cover letter to let us know and include the name of the journal where the work was previously considered and the manuscript ID it was given. In addition, please upload a response to the reviews as a 'Prior Peer Review' file type, which should include the reports in full and a point-by-point reply detailing how you have or plan to address the reviewers' concerns.

During the process of completing your manuscript submission, you will be invited to opt-in to posting your pre-review manuscript as a bioRxiv preprint. Visit http://journals.plos.org/plosbiology/s/preprints for full details. If you consent to posting your current manuscript as a preprint, please upload a single Preprint PDF.

Feel free to email us at plosbiology@plos.org if you have any queries relating to your submission.

Kind regards,

Ines

--

Ines Alvarez-Garcia, PhD

Senior Editor

PLOS Biology

ialvarez-garcia@plos.org

# Peer review: Decision Letter 1

**DOI:** 10.1371/journal.pbio.3003555.r002

**Authors:** Ines Alvarez-Garcia

10 Feb 2026

Dear Elena,

Thank you for your patience while your manuscript entitled "Collagen I promotes cancer cell survival via amino acid import and mTORC1 activation" was peer-reviewed at PLOS Biology. Please also accept my sincere apologies again for the delay in sending you our decision, mostly due to the holidays. The manuscript has now been evaluated by the PLOS Biology editors, an Academic Editor with relevant expertise, and by two independent reviewers.

The reviews are attached below. As you will see, the reviewers find the conclusions interesting and novel, but they also raise several concerns that would need to be addressed before we can consider the manuscript further for publication. Reviewer 1 thinks the study would be significantly strengthen by in vivo knockdown or knockout of the LAT1-4F2hc transporter and assessment of tumour growth in preclinical models using the same cell lines used in vitro. This reviewer also mentions that while the in vitro glucose-starvation system is valuable to identify glucose-specific effects, it doesn’t fully capture the complexity of the tumour microenvironment, thus it should be shown if the alterations are specific to glucose deprivation or if similar changes in amino acid import and mTOR signalling occur under other nutrient-limiting conditions. In addition, the reviewer thinks that data from non-glucose-starved control conditions in the BTT-3033 and siRNA experiments should be included. Reviewer 2 raises several major points, including inconsistent timepoints in the cell growth assays, the link between the alpha2beta1 integrin and mTORC1 activation that is not convincing and that it should be shown if S6 phosphorylation is taking place. The reviewer also mentions that it would be important to show LAT1 mediated amino acid uptake, and confirm that SLC3A2 and SLC7A5 colocalise, among other issues.

In light of the reviews and further discussion with the Academic Editor, we would like to invite you to revise the work to thoroughly address the reviewers' reports. However, we will not insist on the addition of the in vivo experiments recommended by Reviewer 1 for publication in PLOS Biology.

Given the extent of revision needed, we cannot make a decision about publication until we have seen the revised manuscript and your response to the reviewers' comments. Your revised manuscript is likely to be sent for further evaluation by all or a subset of the reviewers.

In addition to these revisions, you will need to complete some formatting changes, which you will receive in a follow up email. A member of our team will be in touch with a set of requests shortly.

We expect to receive your revised manuscript within 3 months. Please email us (plosbiology@plos.org) if you have any questions or concerns, or would like to request an extension.

At this stage, your manuscript remains formally under active consideration at our journal; please notify us by email if you do not intend to submit a revision so that we may withdraw it.

**IMPORTANT - SUBMITTING YOUR REVISION**

Your revisions should address the specific points made by each reviewer. Please submit the following files along with your revised manuscript:

1. A 'Response to Reviewers' file - this should detail your responses to the editorial requests, present a point-by-point response to all of the reviewers' comments, and indicate the changes made to the manuscript.

*NOTE: In your point-by-point response to the reviewers, please provide the full context of each review. Do not selectively quote paragraphs or sentences to reply to. The entire set of reviewer comments should be present in full and each specific point should be responded to individually, point by point.

You should also cite any additional relevant literature that has been published since the original submission and mention any additional citations in your response.

2. In addition to a clean copy of the manuscript, please also upload a 'track-changes' version of your manuscript that specifies the edits made. This should be uploaded as a "Revised Article with Changes Highlighted" file type.

3. Resubmission Checklist

When you are ready to resubmit your revised manuscript, please refer to this resubmission checklist: https://plos.io/Biology_Checklist

To submit a revised version of your manuscript, please go to https://www.editorialmanager.com/pbiology/ and log in as an Author. Click the link labelled 'Submissions Needing Revision' where you will find your submission record.

Please make sure to read the following important policies and guidelines while preparing your revision and fulfil the editorial requests:

a) *PLOS Data Policy*

Please note that as a condition of publication PLOS' data policy (http://journals.plos.org/plosbiology/s/data-availability) requires that you make available all data used to draw the conclusions arrived at in your manuscript. If you have not already done so, you must include any data used in your manuscript either in appropriate repositories, within the body of the manuscript, or as supporting information (N.B. this includes any numerical values that were used to generate graphs, histograms etc.). Please also indicate in each figure legend where the data can be found. For an example see here: http://www.plosbiology.org/article/info%3Adoi%2F10.1371%2Fjournal.pbio.1001908#s5

b) *Published Peer Review*

Please note while forming your response, if your article is accepted, you may have the opportunity to make the peer review history publicly available. The record will include editor decision letters (with reviews) and your responses to reviewer comments. If eligible, we will contact you to opt in or out. Please see here for more details:

https://blogs.plos.org/plos/2019/05/plos-journals-now-open-for-published-peer-review/

c) *Blot and Gel Data Policy*

Please provide the original, uncropped and minimally adjusted images supporting all blot and gel results reported in an article's figures or Supporting Information files. We will require these files before a manuscript can be accepted so please prepare them now, if you have not already uploaded them. Please carefully read our guidelines for how to prepare and upload this data: https://journals.plos.org/plosbiology/s/figures#loc-blot-and-gel-reporting-requirements

d) *Protocols deposition*

To enhance the reproducibility of your results, we recommend that if applicable you deposit your laboratory protocols in protocols.io, where a protocol can be assigned its own identifier (DOI) such that it can be cited independently in the future. For instructions see: https://journals.plos.org/plosbiology/s/submission-guidelines#loc-materials-and-methods

Thank you again for your submission to our journal. We hope that our editorial process has been constructive thus far, and we welcome your feedback at any time. Please don't hesitate to contact us if you have any questions or comments.

Sincerely,

Ines

--

Ines Alvarez-Garcia, PhD

Senior Editor

PLOS Biology

ialvarez-garcia@plos.org

------------------------------------

Reviewers' comments

Rev. 1:

The manuscript by Nazemi et al. describes the role of collagen I as a survival factor for breast and pancreatic cancer cell lines cultured under glucose-starvation conditions. The study is generally well written and easy to follow, and the conclusions are largely supported by the data presented. There are, however, several issues that should be addressed prior to publication to strengthen the impact and clarity of the work.

The authors' data suggest that the LAT1-4F2hc transporter is critical for the growth of breast and pancreatic cancer cells in vitro under glucose-starved conditions, raising the possibility that this transporter may represent a therapeutic target in vivo. While the presented data support this hypothesis, the manuscript would be significantly strengthened by in vivo validation. Specifically, genetic knockdown or knockout of LAT1-4F2hc, followed by assessment of tumor growth in preclinical models using the same cell lines employed in vitro, would provide more compelling evidence for its role in tumor biology.

One limitation of the current experimental design is that tumor cells in vivo experience deprivation of multiple nutrients simultaneously, rather than glucose alone. Although the in vitro glucose-starvation system is valuable for isolating glucose-specific effects, it may not fully capture the complexity of the tumor microenvironment. It would therefore be informative to determine whether the reported alterations are specific to glucose deprivation or whether similar changes in amino acid import and mTOR signaling occur under additional nutrient-limiting conditions, such as reduced serum and/or growth factor availability.

For the BTT-3033 and siRNA experiments, inclusion of data from non-glucose-starved control conditions would be very helpful. Such experiments would clarify whether the observed effects reflect activation of a stress-induced pathway or a constitutively active mechanism independent of glucose deprivation. In particular, while the results suggest that α2β1 integrin modulates cell growth on collagen under glucose-starved conditions, it remains unclear whether similar effects would be observed under nutrient-replete conditions.

Several specific points also warrant clarification:

* Figure 4D: In the bar graph, is the decrease in LC3-II intensity following BTT-3033 and rapamycin treatment statistically significant? This should be clearly indicated.

* Figure 5: Providing the raw metabolic data values represented by the red dots in panels B and E as a supplemental Excel table would improve transparency. The same applies to Figures S8B and S8D.

* Figure 2: Please explicitly state in the main text that the cells were glucose starved in this experiment. Although this is implied by the figure legend title, inclusion in the text would improve clarity for readers.

* Page 5, line 1: The statement that α2β1 is the "main" collagen receptor is not sufficiently supported, given the number of known collagen I receptors. A more precise phrasing, such as "one of the most well-characterized collagen receptors," would be more appropriate.

* Page 20, Methods section - Starvation conditions: The glucose-starvation conditions are described as the use of glucose- and pyruvate-free medium supplemented with 10% dialyzed FBS. This approach suggests that, in addition to glucose deprivation, the removal of pyruvate and other small molecules normally present in serum could also contribute to the observed phenotypes. However, throughout the manuscript, the experimental condition is described primarily as "glucose starvation," which may oversimplify the nature of the nutrient deprivation. The authors are encouraged to clearly define what they mean by glucose starvation early in the main text and to acknowledge that deprivation of additional metabolites may also influence the observed responses in these experiments.

Addressing these points would improve the rigor and interpretability of the study and further enhance its contribution to the field.

Rev. 2: Ersa Gjelaj – note that this reviewer has signed the review

This manuscript aims to address an interesting question, how collagen I supports cancer cell survival under nutrient stress. The observation that collagen I improves survival of invasive cancer cells during glucose deprivation through activation of mTORC1 potentially mediated through LAT1-dependent uptake of amino acids is novel and could be of interest to broad readership. However, the mechanistic model proposed, that collagen I activates ⍺2β1 mediated mTORC1 activation, and either directly, or in parallel, increased LAT1 membrane localisation supports amino acid uptake and mTORC1 signalling to support anabolism, is not convincingly demonstrated. At present, the data show several correlations, but these are weakened by inconsistencies in experimental design that make the data difficult to interpret.

Major Points:

1. Cell growth assays have inconsistent timepoints. Growth curves start at different days depending on the cell line (i.e., MDA MB 231 measured from D2 and PANC1 starting from D0). D2 time points already show significant differences between collagen and plastic, hard to discern whether this is due to inconsistent plating at D0 or a result of increased proliferation. Include D0 measurements. It would also be beneficial to include Complete media conditions here, to see how much collagen rescues proliferation under Glc deprivation.

2. The link between ⍺2β1 and mTORC1 activation is not convincingly demonstrated. Authors show that Collagen increases pS6 and a2B1 inhibition reduces pS6, they also show that this is not the case for 4E-BP1, but there is no actual demonstration that S6 phosphorylation is occurring downstream of mTORC1. Assessing upstream of S6, such as the p70S6K1 as well as blocking mTORC1 with rapamycin/Torin1 will help confirm this. I also strongly suggest assessing mTORC1 using western blots and including both phosphorylated and unphosphorylated downstream targets, to ensure that increased phosphorylation is a result of increased signalling and not protein levels.

3. LAT1 mediated amino acid uptake is not directly demonstrated. They show that there is an increase in amino acid pools on collagen compared to plastic under glucose deprivation. LAT1 subunits show increased surface staining, and knockdown reduces intracellular amino acids. These are measurements of steady-state pools of amino acids, and do not reflect transport rates. While, I am happy to see the authors assessed the contribution of autophagy to these pools, this accumulation could be a result of reduced utilisation due to low proliferation and reduced protein synthesis (likely the case under Glc deprivation). Authors should show increased transport with radiolabelled or stable-isotope labelled AA uptake assays over a dynamic range of time and block LAT1 pharmacologically with a specific inhibitor such as JPH203 for example, or by knockdown.

4. Should co-stain SLC3A2 and SLC7A5 to show that they are co-localised rather than immunostaining individually. Furthermore, it would be good to include complete media conditions here, it seems SLC3A2 is not being trafficked to the membrane efficiently, perhaps due to reduced N-glycosylation as a result of reduced Glc availability.

5. LAT1 localisation and amino acid levels are assessed independently of a2B1 manipulation, unclear how this is specific to collagen under Glc deprivation. Would be beneficial to assess whether a2B1 inhibition alters LAT1 membrane localisation and to assess whether amino acid uptake is altered.

6. SLC3A2 knockdown is not a LAT1 specific perturbation. As mentioned by the authors, SLC3A2 is important for different amino acid transporters including xCT (which is very important under glucose deprivation and potentially here considering Gly/Ser metabolism is a top hit in all untargeted metabolomics studies). Should use SLC7A5 knockdown for mechanistic claims.

7. mTORC1 dependence on LAT1 effects is not tested, they show that SLC3A2 knockdown reduces pS6, but do not test whether LAT1 inhibition reduces collagen-induced mTORC1 activation.

8. Should assess mechanistic claims within short term Glc starvation to avoid the effects of long-term glucose starvation.

9. Overstatements such as "this implies the presence of collagen I alleviates the metabolic stress induced by Glc deprivation enabling cells to maintain an anabolic state". While you see that there is phosphorylation of S6, the dephosphorylation of 4E-BP1 suggests there is no cap-dependent translation, a core anabolic process. You could support rescued anabolism with puromycin incorporation assays to show that global protein translation or ribosome biogenesis with 5-EU incorporation is increased under Glc deprivation with collagen.

10. There is a lack of complete media controls throughout the entire study. I believe these important to understand just how much collagen is protecting cells from glucose deprivation. Similarly in some places where claims are made about collagen there isn't a plastic control.

11. You state that "a2B1 integrin was required for cancer cell growth and invasion under Glc starvation" but you show none of these results under complete media.

12. Figure 7 you show that the a2 and 3A2 KD reduce S6 but you should include the plastic conditions to show that there is first an increase in pS6 and then this reduced to the plastic/complete media conditions.

13. Should prove that D-Phe does block LAT1 transport using an uptake assay.

14. Does D-phe block mTORC1 activation in presence of Coll + Glc deprivation like a2 inhibition?

15. Doesn't seem to be any collagen specific differences in LAT1 requirement, equally required in complete and Glc free media and differ by cell line, but the caption states "SLC3A2 and SLC7A5 were required for collagen I-dependent cell growth in 2D and 3D systems." Should show differences between collagen and plastic.

Minor comments:

- Figure 8F and 3J are duplicated and I can see that the Nt si appears the same in both experiments, this should be highlighted in the caption for Figure 8.

- Validate rapamycin effectiveness in figure 4C/D. Should have downstream targets of mTORC1 from the same lysates to show that rapamycin reduces pS6.

- Is the Rapamycin column in figure C D supposed to be with collagen? To see whether autophagy is increased to plastic levels? Currently "-".

- GAPDH is not a good loading control particularly for metabolic studies as it is influenced by glycolysis and ROS, I recommend replacing these with a different loading control or using total protein staining/ponceau.

- It is hard to understand what cell line some figures are referring to at a glance, would be helpful to clearly label figure panels.

- Please label heatmap scales from metabolomics studies, unclear what they represent.

- Figure 4 A, B don't include PANC1 cells, should keep consistent with the cell lines you established

- Remove overstatements where you only have causal relationships

- Comparative conditions should be on the same graph, Glc starvation and Complete media on different graphs with different scales for Figure 8a/b.

# Author comment: Author response to Decision Letter 2

**DOI:** 10.1371/journal.pbio.3003555.r003

19 Jun 2026

Attachment Submitted filename: Reviewers_response_PBIOLOGY-D-25-03621R1.pdf

# Peer review: Decision Letter 2

**DOI:** 10.1371/journal.pbio.3003555.r004

**Authors:** Ines Alvarez-Garcia

29 Jul 2026

Dear Elena,

Thank you for your patience while we considered your revised manuscript entitled "Collagen I promotes cancer cell survival via amino acid import and mTORC1 activation" for publication as a Research Article at PLOS Biology. This revised version of your manuscript has been evaluated by the PLOS Biology editors, the Academic Editor and the two original reviewers.

Based on the reviews, we are likely to accept this manuscript for publication, provided you satisfactorily address the remaining points raised by Reviewer 2 by moderating the conclusions according to his/her suggestions, but we will not insist on further experiments. Please also make sure to address the policy-related requests stated below my signature.

As you address these items, please take this last chance to review your reference list to ensure that it is complete and correct. If you have cited papers that have been retracted, please include the rationale for doing so in the manuscript text, or remove these references and replace them with relevant current references. Any changes to the reference list should be mentioned in the cover letter that accompanies your revised manuscript.

In addition to these revisions, you may need to complete some formatting changes, which you will receive in a follow up email. A member of our team will be in touch with a set of requests shortly. If you do not receive a separate email within a few days, please assume that checks have been completed, and no additional changes are required.

We expect to receive your revised manuscript within two weeks.

To submit your revision, please go to https://www.editorialmanager.com/pbiology/ and log in as an Author. Click the link labelled 'Submissions Needing Revision' to find your submission record. Your revised submission must include the following:

- a cover letter that should detail your responses to any editorial requests, if applicable, and whether changes have been made to the reference list

- a Response to Reviewers file that provides a detailed response to the reviewers' comments (if applicable, if not applicable please do not delete your existing 'Response to Reviewers' file.)

- a track-changes file indicating any changes that you have made to the manuscript.

NOTE: If Supporting Information files are included with your article, note that these are not copyedited and will be published as they are submitted. Please ensure that these files are legible and of high quality (at least 300 dpi) in an easily accessible file format. For this reason, please be aware that any references listed in an SI file will not be indexed. For more information, see our Supporting Information guidelines:

https://journals.plos.org/plosbiology/s/supporting-information

*Published Peer Review History*

Please note that you may have the opportunity to make the peer review history publicly available. The record will include editor decision letters (with reviews) and your responses to reviewer comments. If eligible, we will contact you to opt in or out. Please see here for more details:

https://plos.org/published-peer-review-history/

*Press*

Should you, your institution's press office or the journal office choose to press release your paper, please ensure you have opted out of Early Article Posting on the submission form. We ask that you notify us as soon as possible if you or your institution is planning to press release the article.

*Protocols deposition*

To enhance the reproducibility of your results, we recommend that if applicable you deposit your laboratory protocols in protocols.io, where a protocol can be assigned its own identifier (DOI) such that it can be cited independently in the future. Additionally, PLOS ONE offers an option for publishing peer-reviewed Lab Protocol articles, which describe protocols hosted on protocols.io. Read more information on sharing protocols at https://plos.org/protocols?utm_medium=editorial-email&utm_source=authorletters&utm_campaign=protocols

Please do not hesitate to contact me should you have any questions.

Sincerely,

Ines

--

Ines Alvarez-Garcia, PhD

Senior Editor

PLOS Biology

ialvarez-garcia@plos.org

------------------------------------------------------------------------

DATA POLICY:

Many thanks for providing all the data underlying the graphs shown in the figures. I have checked all the data and I am missing the data underlying the volcano plot shown in Figure S10B. Please provide the data in the corresponding data file or specify in the figure legend where the data can be found.

------------------------------------------------------------------------

CODE POLICY

Per journal policy, if you have generated any custom code during the course of this investigation, please make it available without restrictions. Please ensure that the code is sufficiently well documented and reusable, and that your Data Statement in the Editorial Manager submission system accurately describes where your code can be found. More information on our Code Policy, what and how to share can be found here: https://journals.plos.org/plosbiology/s/code-availability

Please note that we cannot accept sole deposition of code in GitHub, as this could be changed after publication. However, you can archive this version of your publicly available GitHub code to Zenodo. Once you do this, it will generate a DOI number, which you will need to provide in the Data Accessibility Statement (you are welcome to also provide the GitHub access information). See the process for doing this here: https://docs.github.com/en/repositories/archiving-a-github-repository/referencing-and-citing-content

------------------------------------------------------------------------

Reviewers' comments

Rev. 1:

The authors have satisfactorily addressed my concerns.

Rev. 2:

I appreciate the changes the authors have made to address my previous concerns, however, some concerns still remain.

Major comments:

1. I previously requested a more direct demonstration of LAT1-mediated AA transport because the targeted metabolomics experiments measure "steady-state" intracellular amino acid pools, which can reflect changes in transport, metabolism, protein synthesis, etc. I appreciate that the authors have included a dynamic 13C-Tyr uptake exp using D-Phe. However, the reduction in 13C-Tyr uptake is relatively modest, making it difficult to conclude that LAT1 import is efficiently blocked. Furthermore, the uptake assay does not directly compare AA transport between collagen vs. plastic or complete vs. Glc-starved conditions, which is central to the proposed model. While SLC7A5 KD demonstrates that LAT1 contributes to intracellular AA levels it again is not compared to complete media conditions, is there actually increased import relative to complete conditions in Coll-I seeded cells or is it maintained? Therefore, I do not think the current data fully support that collagen I promotes EAA import under Glc- starvation. Either stronger transport experiments should be provided (i.e., using a more selective LAT1 inhibitor like JPH203, or additional substrate competition rescues with D-Phe) with the necessary control conditions, or conclusions should be moderated throughout the manuscript to state that coll I increased intracellular EAA abundance (if complete media + Coll I metabolite experiments are done). Additionally, can the authors clarify what the targeted metabolomics experiments are normalized to.

2. Conclusions regarding mTORC1 activation should be revisited. The additional rapamycin experiments strengthen the conclusion that collagen-induced S6 phosphorylation is largely mTORC1-dependent. Therefore, there are some statements throughout the manuscript that refer to "mTORC1 activation" that should be changed to RPS6 phosphorylation specifically since p70S6K1 and 4E-BP1 were not altered in Coll I conditions. Furthermore, the conclusion that "mTORC1 signalling was required for ECM-dependent cell growth under Glc-" is not fully supported by the current experiments. Figure 4D/E only examines Rapa treatment in collagen-grown Glc-starved cells and not complete medium and on plastic, therefore, it doesn't demonstrate that it's required for ECM-dependent growth. Including the corresponding controls would clarify how much of the collagen phenotype is mTORC1-dependent.

3. Mechanistic interpretation of SLC3A2 KD should be moderated. The manuscript states that SLC3A2 KD demonstrates that "LAT1-4f2hc transporter mediated coll-I dependent mTORC1 activation and autophagy inhibition". SLC3A2 is not specific to LAT1 so SLC3A2 KD results cannot be attributed solely to LAT1 activity. I recommend moderating these statements relating to Fig 8 unless experiments are repeated with the SLC7A5 KD cells generated in Fig 7H,I.

4. The targeted metabolomics performed after 4 days of Glc starvation should be interpreted cautiously. By this stage, Fig 1 demonstrates substantial cell death in cells grown on plastic, whereas Coll-I grown cells remain largely viable. Consequently, metabolic differences may reflect differences in cell survival rather than metabolic differences. It would also be helpful to include the Day 1 and Day 2 cell death quantification for these cell lines (Fig 1E), as these correspond to the time points analysed in Figures 6G/H.

Minor comments:

1. Why were spheroid experiments performed for different durations (2 days for MDA-MB-231 vs. 6 days for PANC1)? A brief justification would be helpful.

2. Please include WB validation for SLC7A5 KD efficiency.

3. For the serum starvation experiments (Figure S15), was the ISR activated to a similar extent as during Glc starvation? In addition, how was the 8 day serum starvation timepoint selected?

# Author comment: Author response to Decision Letter 3

**DOI:** 10.1371/journal.pbio.3003555.r005

26 Aug 2026

Attachment Submitted filename: Reviewers_response_PBIOLOGY-D-25-03621R2.pdf

# Editor report: Decision Letter 3

**DOI:** 10.1371/journal.pbio.3003555.r006

**Authors:** Ines Alvarez-Garcia

6 Sep 2026

Dear Elena,

Thank you for the submission of your revised Research Article entitled "Collagen I promotes cancer cell survival via amino acid import and mTORC1/S6 activation" for publication in PLOS Biology. On behalf of my colleagues and the Academic Editor, Albana Gattelli, I am delighted to let you know that we can in principle accept your manuscript for publication, provided you address any remaining formatting and reporting issues. These will be detailed in an email you should receive within 2-3 business days from our colleagues in the journal operations team; no action is required from you until then. Please note that we will not be able to formally accept your manuscript and schedule it for publication until you have completed any requested changes.

Please take a minute to log into Editorial Manager at http://www.editorialmanager.com/pbiology/, click the "Update My Information" link at the top of the page, and update your user information to ensure an efficient production process.

PRESS

We frequently collaborate with press offices. If your institution or institutions have a press office, please notify them about your upcoming paper at this point, to enable them to help maximise its impact. If the press office is planning to promote your findings, we would be grateful if they could coordinate with biologypress@plos.org. If you have previously opted in to the early version process, we ask that you notify us immediately of any press plans so that we may opt out on your behalf.

We also ask that you take this opportunity to read our Embargo Policy regarding the discussion, promotion and media coverage of work that is yet to be published by PLOS. As your manuscript is not yet published, it is bound by the conditions of our Embargo Policy. Please be aware that this policy is in place both to ensure that any press coverage of your article is fully substantiated and to provide a direct link between such coverage and the published work. For full details of our Embargo Policy, please visit http://www.plos.org/about/media-inquiries/embargo-policy/.

Many congratulations and thanks again for choosing PLOS Biology for publication and supporting Open Access publishing. We look forward to publishing your study.

Sincerely,

Ines

--

Ines Alvarez-Garcia, PhD

Senior Editor

PLOS Biology

ialvarez-garcia@plos.org
